# Post-hysterectomy rare collision vulva tumor with long-term human papilloma virus infection composed of squamous cell carcinoma of the labia major and adenosquamous carcinoma of bartholin gland: A case report

**DOI:** 10.1097/MD.0000000000017043

**Published:** 2019-09-27

**Authors:** Fan Yang, HongYi Li, Xiaorong Qi, Ce Bian

**Affiliations:** a Department of Obstetrics & Gynaecology, West China Second Hospital; b Key Laboratory of Obstetric & Gynaecologic and Pediatric Diseases and Birth Defects of Ministry of Education, Sichuan University, Chengdu, Sichuan, China.

**Keywords:** bartholin gland, collision tumor, human papilloma virus infection, post-hysterectomy, vulva

## Abstract

**Rationale:**

Post-hysterectomy collision tumors of the vulva has rarely been reported. Though long-term HPV infection may induce vulva tumor, but the relationship between HPV infection and collision vulva tumor is not clear. And there are no clear rules of the post-hysterectomy cancer surveillance for human papilloma virus (HPV) long-term infections. So here we first report a case of post-hysterectomy rare collision vulva tumor with long-term HPV infection composed of squamous cell carcinoma of the labia major and adenosquamous carcinoma of bartholin gland and hope to bring new direction to our future research.

**Patient concerns:**

A 48-year-old woman with long-term HPV infection, 3 years after hysterectomy, gravida 3, para 2, was admitted to our hospital with complaints of a 4-month history of an itching vulva ulceration. An anabrosis was located on the surface of the solid mass of the bartholin gland at the posterior part of the right labium and the right inguinal lymph nodes were palpable. Result of the incisional biopsy of the ulcer area at local hospital was atypical squamous cells couldn’t exclude high-grade squamous intraepithelial lesion (ASC-H). Subsequently more authoritative pathological consultation results suggested squamous cell carcinoma of the vulva.

**Diagnoses:**

Post-hysterectomy collision vulva tumor with long-term HPV infection composed of squamous cell carcinoma of the labia major and adenosquamous carcinoma of bartholin gland.

**Interventions:**

The extensive excision of the vulva, bilateral inguinal lymph nodes dissection, and local skin flap transposition surgeon was done to this patient. The final certificate diagnosis was: vulvar tumor T1bM0N0 composed of squamous cell carcinoma of the labia major and adenosquamous carcinoma of bartholin gland; HPV infection; post hysterectomy, and bilateral salpingectomy.

**Outcomes:**

The patient recovered well after surgery, and consequently received 6 courses of TC (paclitaxel + carboplatin) chemotherapy, and 9 months and 13 days followed up. So far patient recorded as complete response (CR).

**Lessons:**

Collision vulva tumor occurred post-hysterectomy is extremely rare. It is most likely related to long-term HPV infection, which suggests us should to modify the manner of the post-hysterectomy cancer surveillance for HPV long-term infections. For patients with high-risk HPV infection, even if the cytology results are negative, we may should perform colposcopy and vulva biopsy more positively to prevent the disease from progressing into cancer. And the pathogenesis of relationship between HPV infection and collision vulva tumor is still need further investigation.

## 1 Introduction

Vulvar cancer accounts for 5% of all gynecologic cancers with an incidence of 2.5 per 100,000 women.^[1]^ Epidemiologically, the most common histological type of vulvar cancer is non-Bartholin gland-related vulvar squamous cell carcinomas (SCCs), which contributes to 80% of worldwide vulva cancer cases. While adenosquamous carcinoma of bartholin gland is rare forms of vulvar malignancy, which are rarely reported and account for <2% of cases.^[2]^ Collision tumor are exceedingly rare in the vulva, with only two cases reported in the English literature, associated with squamous cell carcinoma^[3]^ or adenocarcinoma.^[4]^

Human papilloma virus (HPV) infection has been reported to have a role in the pathogenesis of post-hysterectomy vaginal and vulvar dysplasia.^[5]^ It is found that in post-hysterectomy HPV prevalence was higher among vaginal than vulvar cases, and HPV16 accounted for most HPV-positive cases for both cancers. Colposcopy examination can detect most preneoplastic lesions, whereas HPV genotyping is suggested to be a more sensitive, inexpensive, and noninvasive method for diagnosis.^[6]^

However, data of in post-hysterectomy on vulvar cancers are limited. We here first report a post-hysterectomy collision vulva tumor, who had long-term HPV infection, composed of squamous cell carcinoma of the labia major and adenosquamous carcinoma of bartholin gland.

## 2 Case report

**Figure 1:**
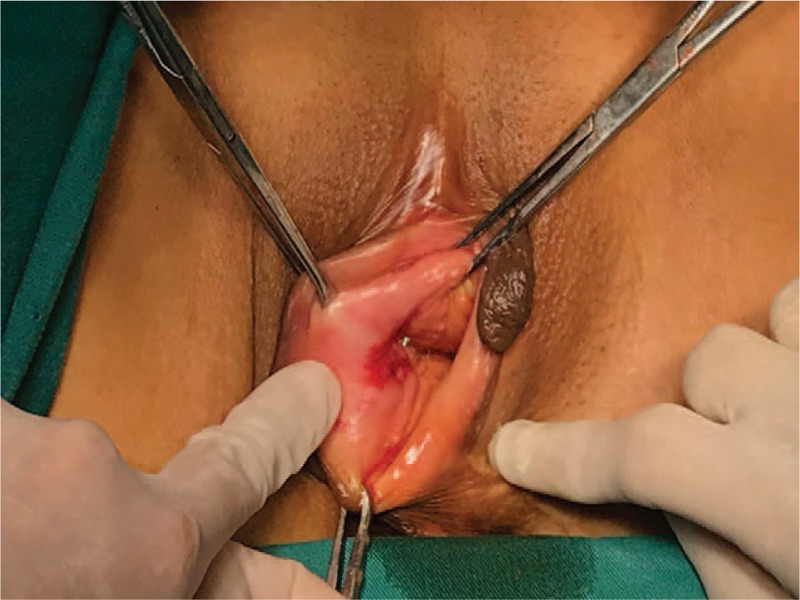
Photo of the collision tumor. a 2.0 cm × 1.5 cm tumor with a well-defined, expansile margin on the posterior part of the right labium and a 3.0 cm × 2.0 cm well-circumscribed, solid mass of the bartholin gland that is unilocular cyst filled with mucoid material and a hemorrhagic lining, without papillary or polypoid projections was seen. The patient underwent extensive excision of the vulva, bilateral inguinal lymph nodes dissection, and local skin flap transposition.

A 48-year-old post-hysterectomy woman with long-term HPV infection, gravida 3, para 2 (G3P2), was referred to our hospital with a 4-month history of an itching vulva ulceration. The patient was diagnosed as grade III of cervical intraepithelial neoplasia with HPV-16 infection. In June 2015, and underwent laparoscopic hysterectomy and bilateral salpingectomy at their local hospital. After the surgery her follow-up revealed a long-term vaginally HPV-16 infection but negative vaginal cytology. At admission, the clinical examination demonstrated 2.0 cm × 1.5 cm sized anabrosis was located on the posterior part of the right labium on a 3.0 cm × 2.0 cm well-circumscribed solid mass of the bartholin gland (Fig. 1). Enlarged lymph nodes of the right inguinal were palpable, and the pelvic was empty owing to her excision of bilateral accessory and hysterectomy for a history of cervical intraepithelial neoplasia III (CIN III) 3 years ago. The result of HPV testing detection of the vaginal discharge at the time of present admission suggests that type HPV-16 and HPV-59 are positive, and the remaining subtypes were negative, while the screen of vaginal cytology smear showed negatively. An incisional biopsy of the ulcer area was performed at local hospital and initially diagnosed as atypical squamous cells couldn’t exclude high-grade squamous intraepithelial lesion (ASC-H). Subsequently a more authoritative pathological consultation results suggested as a squamous cell carcinoma of the vulva. No abnormality was found by transvaginal ultrasonography but pelvic abdominal computed tomography showed absent uterus and slightly thickened vaginal cuff with increased inguinal lymph nodes. Preoperatively, the tumor markers such as squamous cell carcinoma antigen was 1.6 ng/mL, and CA 125 and CEA were within normal limit.

The patient underwent extensive excision of the vulva, bilateral inguinal lymph nodes dissection, and local skin flap transposition. Vulvar cancer of this case was staged as T_1b_M_0_N_0_ using the American Joint Committee on Cancer (AJCC) and the International Federation of Gynecology and Obstetrics (FIGO) staging systems.

**Figure 2:**
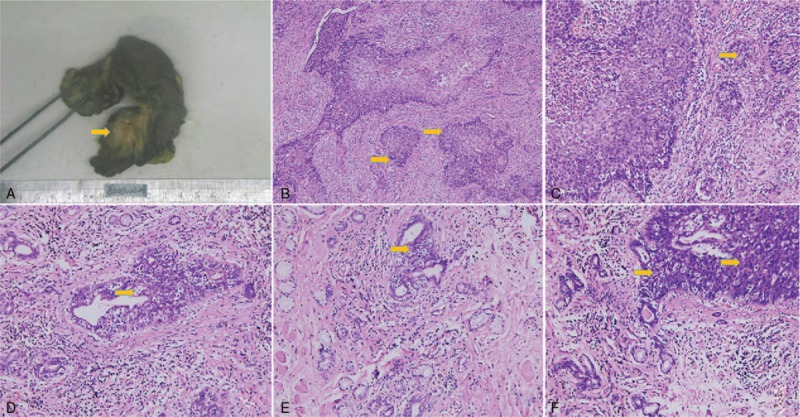
Postoperation photo of pathological specimen on microscope. A, The gross postoperative pathology. It illustrated a 2.0 cm × 1.5 cm tumor was found with a well-defined, expansile margin on the posterior part of the right labium; meanwhile another solid mass of the bartholin gland was found which was a 3.0 cm × 2.0 cm well-circumscribed unilocular cyst filled with mucoid material and a hemorrhagic lining, without papillary or polypoid projections in. B and C, The squamous cell carcinoma of the labia major. The HE staining of squamous cell carcinoma of the labia majora. B is under 10 times light microscope, and C is under 40 times light microscope. The cells are arranged in disorder, the cell size is different, the cell is heterogeneous, there is a pathological mitotic phase, and keratinized beads appear, showing an intercellular bridge. The lesions are labeled by arrows in the figures. C–E, The adenosquamous carcinoma of bartholin gland. They are under 40 times light microscope, squamous cell carcinoma, and adenocarcinoma components and intermediate components can be seen in C andE, tumor thrombus around the vessel can be found in D. The lesions are labeled by arrows in the figures.

The postoperation histopathological results certificated the diagnosis as: collision vulva tumor with long-term HPV infection composed of squamous cell carcinoma of the labia major and adenosquamous carcinoma of bartholin gland. The gross postoperative pathology description illustrated, a 2.0 cm × 1.5 cm tumor was found with a well-defined, expansile margin on the posterior part of the right labium; meanwhile another solid mass of the bartholin gland was found which was a 3.0 cm × 2.0 cm well-circumscribed unilocular cyst filled with mucoid material and a hemorrhagic lining, without papillary or polypoid projections in. Subsequently on microscopic examination, it indicated, the tumor of the large labia was a 2.0 cm × 1.5 cm × 1.0 cm poor differentiated squamous cell carcinoma and appeared to be submucosal invasion with infiltration depth approximately reaching 1.0 cm. Intravascular tumor thrombus were found and extensive vaginal intraepithelial lesions II to III existed in peri carcinomatous tissue. Simultaneously, the solid mass of bartholin gland revealed to be completely another poorly differentiated adenosquamous carcinoma with partial undifferentiated element (Fig. 2). Immunohistochemistry had been done respectively in excision tumors to verify our judgment. Results showed diffuse positive for CK5/6, P63, P40, and P16 in the squamous cell carcinoma component with 85% positive rate of Ki67. However, the adenosquamous carcinoma components of bartholin gland were positive for CD10, VIM, and focal positive for CAM5.2, CK5/6. The other tumor markers like CEA, ER, PR, and Muc-2- were all negative for both (details are shown in Table 1). Meanwhile, DNA detection of high-risk HPV types were repeated, HPV-16, and HPV-59 were positive for both. The surgical margins and inguinal lymph nodes were histologically unremarkable without cancer metastasis. The final certificate diagnoses on discharge were: vulvar tumor T1bM0N0 composed of squamous cell carcinoma of the labia major and adenosquamous carcinoma of bartholin gland; HPV infection; post hysterectomy and bilateral salpingectomy.

**Table 1:**
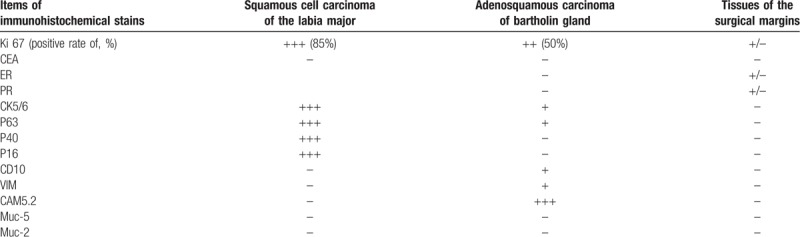
Postoperative immunohistochemical stains used for differentiating the vulvar tumor.

The patient was discharged after 12 days of hospitalization. Chemotherapy was initiated in our hospital tumor center as soon as her surgical incision achieved healing 4 weeks after the surgery. According to the National Comprehensive Cancer Network (NCCN) Clinical Practice Guidelines in Oncology of vulvar cancer, the patient received 6 courses of TC (paclitaxel + carboplatin) chemotherapy: paclitaxel 135 mg/m^2^ + platinum (AUC, the average area under curve of carboplatin) = 4. As a follow-up, the patient underwent a monthly clinical evaluation, including testing of various blood biochemical and tumor marker levels; and the imaging examinations every 3 months, including vaginal ultrasound, magnetic resonance imaging (MRI) or positron emission tomography–computed tomography (PET-CT). From the end of the operation to June 30th, 2019, totally 9 months and 13 days were followed up. So far patient follow-up status has been recorded as complete response (CR) according both to response evaluation criteria in solid tumors (RECIST 1.1), and modified response evaluation criteria in solid tumors (mRECIST), and no recurrence was found.

## 3 Discussion

In this case we first report a post-hysterectomy collision vulva tumor, who had long-term HPV infection, composed of squamous cell carcinoma of the labia major and adenosquamous carcinoma of bartholin gland. Though the squamous cell carcinoma of large labia and adenosquamous carcinoma of bartholin gland are 2 common histological types respectively in vulva, they are exceedingly rare to be found together.

Back to the patient's medical history, we found that long-term HPV infection was an obviously high-risk throughout the course of disease. According to our current knowledge, the patient suffered CINIII 3 years ago, infection of HPV-16 could be the cause of the disease; however, during the follow-up of the hysterectomy, the patient's HPV infection was not cleared but had persisted till now. Therefore, it is reasonable to infer if there is proposal relationship between the collision tumor of vulva and the long-term HPV infections of post-hysterectomy. It has been reported that a single carcinogenic agent may interact with 2 neighboring tissues, inducing the development of tumors of different histological types (epithelial and stromal) in the same organ.^[7]^ Therefore, we hypothesize that the presence of vulva collision tumors may not be purely coincidental, but because of that the vulva was stimulated by a same carcinogen, resulting in a simultaneous proliferation of different cancer cell lines, and the carcinogen may should be HPV infections. Of course, our assumptions are supported by some evidences. It has already been reported that there is some correlation between the occurrence of squamous cell carcinoma of vulva and long-term infection of HPV.^[2,5]^ However, adenosquamous carcinoma of bartholin gland, as one of the rare forms of vulvar malignancy, its association with high-risk HPV infection is still unclear, although there have been some reports about the etiologic role of HPV in the pathogenesis of squamous cell carcinomas (SCC) of bartholin gland. All in all, there might be proposal relationship between long-term HPV infections of post-hysterectomy and the collision tumor of vulva, which requires our further investigations.

Lessons that this case brings to us from another side of the coin is about the post-hysterectomy cancer surveillance. Although this patient has been undergoing vaginal cytology and HPV DNA monitoring every year after hysterectomy, the long-term HPV infection have been ignoring because her vaginal cytology results have been negative. Eventually biopsy confirmed the occurrence of vulvar cancer until the symptom of her vulvar ulceration occurred. Looking back at the entire course of the disease, the long-term high-risk HPV infections of patient did not receive enough attention and vigilance. We reviewed relevant guidelines and literatures on vulvar tumors and found that HPV infection is a high-risk factor for cervical, vaginal, and vulvar tumors.^[8–10]^ Guidelines clearly suggest that for patients with long-term high-risk type infection of HPV in cervical and vaginal secretions, such as HPV-16 or HPV-18, colposcopy should be added, and biopsy of cervical and vaginal tissues should be done if any suspicions, no matter what result of the cytology smear screening is. By further review of the literature on post-hysterectomy cancer surveillance, we found that cervical screening and the histology test of the cervix are recommended for women with a past history of high grade squamous intraepithelial lesions of the cervix (CINII-III).^[11]^ Colposcopy and further vulvar tissue biopsy should be done if having suspicion, especially for long-term HPV infections even without vaginal dysplasia.^[12]^ However, there is no literature mentioned that whether the vulvar tissue should be also focused, and if it is necessary for biopsy of vulvar tissue in the case of negative cytology? The current manner of cancer surveillance might be just the limitation of this case, by which long-term HPV infection has been neglected and eventually developed into a cause of vulvar tumors.

In conclusion, this was an extremely rare collision vulva tumor occurred after hysterectomy. Its occurrence is most likely related to long-term HPV infection. This tragedy suggests us should to modify the manner of the post-hysterectomy cancer surveillance for HPV long-term infections. For patients with high-risk HPV infection, even if the cytology results are negative, we should perform colposcopy and vulva biopsy to prevent the disease from progressing into cancer. At the same time, the pathogenesis of relationship between HPV infection and collision vulva tumor is still need further investigation.

## Author contributions

**Funding acquisition:** Xiaorong Qi.

**Investigation:** Fan Yang, HongYi Li, Xiaorong Qi.

**Methodology:** Ce Bian.

**Resources:** Fan Yang, Ce Bian.

**Software:** HongYi Li.

**Writing – original draft:** Fan Yang.

**Writing – review & editing:** Fan Yang.
